# A review of the impact of circadian rhythm on motor function in stroke recovery patients

**DOI:** 10.3389/fneur.2026.1740734

**Published:** 2026-06-22

**Authors:** Hao Zhou, Tong Zhang, Bingjie Li, Jun Zhao

**Affiliations:** 1 School of Rehabilitation, Capital Medical University Country, Beijing, China; 2 Beijing Bo'ai Hospital, China Rehabilitation Research Center, Beijing, China

**Keywords:** circadian rhythm, motor function, rehabilitation, research technology, stroke

## Abstract

Stroke is a leading cause of motor impairment, significantly affecting patients’ quality of life during the recovery phase. Motor function recovery after stroke is influenced by multiple factors, among which circadian rhythm have recently garnered attention due to their potential role in neurorehabilitation. Circadian rhythm, encompassing circadian and ultradian cycles, regulate various physiological processes and may influence neural plasticity and motor control mechanisms critical for rehabilitation outcomes. Current researches highlight the mechanisms by which circadian rhythm interact with brain function, including modulation of neural feedback loops and brainwave activity, which are essential in restoring motor abilities post-stroke. Despite emerging evidence, challenges remain in fully elucidating the complex relationship between circadian rhythm and motor recovery, particularly in translating these findings into clinical practice. This review synthesizes recent advances in understanding how circadian rhythm affect motor function recovery in stroke patients, and provides an overview of neurofeedback and electroencephalographic modulation techniques. By analyzing current studies and integrating multidisciplinary insights, this review aims to provide a comprehensive perspective on the role of circadian rhythm in stroke rehabilitation, offering novel approaches to enhance motor recovery and improve patient outcomes.

## 1 Introduction

Stroke remains a leading cause of long-term disability worldwide, with motor impairment being one of its most prevalent and debilitating consequences. Motor deficits following stroke severely limit patients’ mobility and their ability to perform activities of daily living, thereby profoundly diminishing quality of life and imposing substantial socioeconomic burdens on individuals and healthcare systems alike (1). Despite advances in conventional rehabilitation approaches, the recovery of motor function remains incomplete for many patients, highlighting an urgent need for novel therapeutic strategies that can more effectively promote neuroplasticity and functional restoration (1, 2). Traditional rehabilitation methods, while beneficial, often face limitations such as therapist and patient fatigue, insufficient intensity or specificity of training, and lack of sustained patient motivation, which can hinder optimal recovery outcomes (3, 4). Consequently, exploring innovative interventions that leverage emerging insights into brain function and neurophysiology is critical for improving stroke rehabilitation paradigms.

Among the promising avenues of research is the investigation of circadian rhythm-endogenous oscillatory patterns of neural activity-that regulate brain function and behavior. Circadian rhythm, particularly brain oscillations detectable via electroencephalography (EEG), reflect coordinated neuronal network dynamics that underpin motor control, sensorimotor integration, and cognitive processes (5). Alterations in these rhythms following stroke may contribute to motor deficits and influence recovery trajectories. For instance, changes in sensorimotor rhythms (SMRs) activity and functional connectivity within motor networks have been associated with impaired motor performance and rehabilitation outcomes (6, 7). Understanding how these rhythms modulate motor function and plasticity could inform the development of neuromodulatory and neurofeedback interventions tailored to individual patients’ neurophysiological profiles.

In particular, EEG-derived metrics such as event-related desynchronization/synchronization (ERD/ERS) in mu and beta frequency bands serve as biomarkers of motor cortex engagement during motor execution, motor imagery, and action observation-processes integral to motor learning and recovery (8, 9). Moreover, dynamic changes in brain connectivity patterns, including the variability and temporal configuration of functional networks, have demonstrated predictive value for motor impairment severity and recovery potential post-stroke (7). These findings underscore the potential of circadian rhythm not only as diagnostic and prognostic tools but also as targets for rehabilitation strategies that harness neuroplastic mechanisms.

This review aims to comprehensively examine the influence of circadian rhythm on motor ability in patients recovering from stroke. We will explore the underlying neurophysiological mechanisms by which oscillatory brain activity and connectivity patterns affect motor function, analyze how disruptions in these rhythms relate to motor impairment severity, and evaluate current and emerging clinical applications that leverage modulation of circadian rhythm to enhance motor recovery. By synthesizing evidence from neuroimaging, electrophysiology, and rehabilitation studies, this article seeks to highlight the critical role of circadian rhythm in post-stroke motor rehabilitation and to identify future directions for research and clinical practice that may improve functional outcomes for stroke survivors.

## 2 Circadian rhythm, stroke and research technology

### 2.1 Basic concepts of circadian rhythm

#### 2.1.1 Definition and classification of circadian rhythm

Circadian rhythm refer to the periodic fluctuations in physiological and behavioral activities within living organisms, orchestrated by endogenous timing systems that align internal functions with external environmental cycles (10). These rhythms are fundamental to maintaining homeostasis and optimizing organismal function across diverse biological scales, from molecular to systemic levels. The most extensively studied category is the circadian rhythm, characterized by approximately 24-h cycles that synchronize physiological processes such as sleep–wake patterns, hormone secretion, metabolism, and cellular functions with the day-night environmental cycle. Circadian rhythms are governed by an internal biological clock, centrally located in mammals within the suprachiasmatic nucleus (SCN) of the hypothalamus, which integrates external cues-primarily light-to entrain internal timing to the environment (11, 12). This central clock communicates with peripheral clocks distributed throughout various tissues and organs, including the liver, muscle, and skin, coordinating systemic rhythmicity (13, 14).

Beyond circadian rhythms, circadian rhythm encompass ultradian rhythms, which occur at intervals shorter than 24 h (e.g., cycles of hormone release or neuronal firing patterns), and infradian rhythms, which span periods longer than a day, such as menstrual cycles or seasonal breeding (15, 16). Ultradian rhythms, while less understood, exhibit distinct regulatory mechanisms and functional roles compared to circadian rhythms, often lacking discrete oscillators and biochemical buffering, which may confer adaptive advantages in metabolic and behavioral regulation (15). The classification of circadian rhythm is thus based on their periodicity: ultradian (<24 h), circadian (~24 h), and infradian (>24 h), each contributing uniquely to physiological regulation.

At the molecular level, circadian rhythm are generated and maintained by transcriptional-translational feedback loops involving core clock genes such as CLOCK, BMAL1, PER, and CRY, which regulate rhythmic gene expression and downstream physiological processes (13, 17). These molecular oscillators are modulated by environmental zeitgebers, with light being the primary synchronizer of circadian clocks. Disruption of these rhythms, termed chronodisruption, can lead to adverse health outcomes, including metabolic disorders, neurodegenerative diseases, and impaired recovery after injury (18, 19).

In summary, circadian rhythm constitute a hierarchy of endogenous oscillations classified primarily by their periodicity into ultradian, circadian, and infradian rhythms. The circadian rhythm, with its approximately 24-h cycle, is the most prominent and extensively characterized, driven by molecular clocks that coordinate physiological and behavioral functions in alignment with environmental cycles. Understanding these rhythms and their classifications provides a crucial framework for exploring their impact on health and disease, including their influence on recovery processes such as post-stroke motor function.

#### 2.1.2 Physiological mechanisms of circadian rhythm

Circadian rhythm, encompassing ultradian, circadian, and infradian cycles, are fundamental to the temporal organization of physiological processes in living organisms. These rhythms are governed by endogenous biological clocks, primarily the circadian clock, which orchestrates a wide array of biochemical, physiological, and behavioral functions in alignment with environmental cycles such as the light–dark (LD) cycle (16, 20). The central circadian pacemaker in mammals resides in the SCN of the hypothalamus, which integrates external cues like photoperiod and synchronizes peripheral clocks across tissues (21). The molecular machinery of the circadian clock involves a transcription-translation feedback loop (TTFL) comprising core clock genes and proteins such as BMAL1, CLOCK, PERs, and CRYs, which generate oscillations with a near 24-h period (22). These clock components regulate rhythmic gene expression that modulates physiological pathways, including hormone secretion, metabolism, immune function, and neuronal excitability (23, 24).

Neurotransmitters and hormones exhibit circadian fluctuations that are integral to the maintenance and output of circadian rhythm. Monoamines such as dopamine, serotonin, and histamine, as well as neuropeptides, participate in the modulation of circadian timing and downstream physiological effects (25). For instance, serotonin influences circadian phase resetting and mood regulation, while dopamine modulates motor activity rhythms and reward processing. The SCN itself exhibits rhythmic neuronal firing patterns and neurotransmitter release, which propagate timing signals to other brain regions and peripheral organs (26). Hormonal rhythms, including those of melatonin, cortisol, and growth hormone, are tightly linked to circadian control and serve as both outputs and feedback regulators of the biological clock (18, 19). Melatonin secretion by the pineal gland peaks during the night and conveys photoperiodic information, reinforcing the synchronization of circadian rhythms (21). Cortisol follows a robust diurnal rhythm with a peak in the early morning, modulating metabolism and stress responses (19). Disruptions in these neuroendocrine rhythms can lead to pathophysiological consequences, including metabolic disorders, cardiovascular diseases, and neuropsychiatric conditions (27, 28).

The interplay between neurotransmitters, hormones, and the molecular clock is bidirectional. CLOCK genes regulate the expression and activity of enzymes involved in neurotransmitter synthesis and degradation, while neurotransmitters can modulate CLOCK gene expression and neuronal activity within the SCN and other circadian centers (29). For example, retinoic acid, a bioactive form of vitamin A, influences circadian rhythms by interacting with nuclear receptors that affect CLOCK gene transcription (29). Additionally, reactive oxygen species and antioxidant systems display circadian variation and may participate in clock regulation (30). Ultradian rhythms, shorter than 24 h, such as the 90-min sleep cycle and hypothalamic neuronal oscillations, are nested within the circadian framework and involve neurotransmitter dynamics and ion channel activity (31, 32).

Environmental factors such as light exposure, feeding times, and physical activity act as zeitgebers (time-givers) that entrain the biological clock and modulate neurotransmitter and hormonal rhythms (27, 33). Exercise, in particular, can influence circadian phase and amplitude by altering hormonal secretions and neurotransmitter release, thus serving as a non-photic entrainment cue (27, 34). The precise timing of these environmental cues is crucial, as mistimed stimuli can disrupt the synchronization of circadian rhythm and impair physiological homeostasis (35).

In summary, circadian rhythms are orchestrated by endogenous clocks that regulate and are regulated by complex neurochemical and hormonal networks. These rhythms ensure temporal coordination of physiological functions, enabling organisms to anticipate and adapt to environmental changes. Understanding the physiological mechanisms underlying circadian rhythm is essential for developing chronotherapeutic strategies to optimize health and recovery, including in clinical contexts such as stroke rehabilitation and metabolic disease management (27, 35).

### 2.2 Biological basis of stroke

#### 2.2.1 Pathophysiology of stroke

Stroke, particularly ischemic stroke, involves a complex cascade of events leading to neural injury and motor dysfunction due to reduced cerebral blood flow, resulting in excitotoxicity, oxidative stress, inflammation, and the activation of immune cells that exacerbate neuroinflammation (36). The ischemic core suffers rapid necrosis, while the surrounding penumbra may be salvageable with timely reperfusion, although it can still experience selective neuronal loss, affecting recovery (37). Additionally, oxidative stress disrupts the balance in brain cells, stroke alters brain oscillations and connectivity, and the immune response influences progression and recovery. Secondary effects like muscle wasting and molecular mechanisms exacerbating neuronal injury contribute to varying degrees of motor impairment, emphasizing the need for targeted therapies and personalized approaches to optimize recovery (38).

#### 2.2.2 Neuroplasticity in the recovery process

Neuroplasticity, defined as the brain’s ability to reorganize and form new neural connections, is fundamental to functional recovery following stroke. After a cerebrovascular event, the brain undergoes a dynamic state of plasticity characterized by neurogenesis, synaptogenesis, axonal sprouting, and cortical reorganization, which collectively contribute to the restoration of motor and cognitive functions impaired by ischemic injury. The significance of neuroplasticity in stroke rehabilitation is underscored by evidence showing that the extent and timing of plastic changes correlate with the degree of motor recovery (39). However, stroke-induced damage can alter the intrinsic mechanisms of neuroplasticity, necessitating interventions that can enhance or restore these processes. Circadian rhythm, especially the circadian and ultradian cycles, is increasingly recognized as a pivotal influencer of neuroplasticity, affecting neuronal excitability, synaptic strength, and the gene expression patterns that facilitate neural remodeling.

Environmental enrichment paradigms, which inherently incorporate rhythmic sensory and motor stimuli, have been shown to promote neuroplasticity and functional recovery post-stroke by modulating epigenetic regulators such as histone deacetylase 2 (HDAC2). Specifically, environmental enrichment reverses stroke-induced HDAC2 upregulation, a negative regulator of neuroplasticity, thereby enhancing the expression of neurotrophins and proteins essential for synaptic plasticity and axonal rewiring (40). This suggests that rhythmic environmental inputs aligned with circadian rhythm can facilitate neuroplastic processes critical for recovery.

Neurorehabilitation strategies that leverage the timing of circadian rhythm may optimize neuroplasticity. For instance, repetitive transcranial magnetic stimulation (rTMS), when combined with aerobic exercise, has demonstrated synergistic effects on motor recovery and neuroplasticity markers such as brain-derived neurotrophic factor (BDNF) signaling pathways in animal models of ischemic stroke (41). The efficacy of such interventions may be enhanced by synchronizing stimulation and training with endogenous circadian rhythm, thereby maximizing cortical excitability and plasticity.

Macrostructural neuroplasticity within motor control networks, including the cerebellum, has been correlated with individual differences in motor recovery, indicating that neuroplastic changes are not only molecular but also involve volumetric and connectivity alterations that may be modulated by rhythmic activity patterns (42). Furthermore, multisensory brain-computer interface (BCI) systems that integrate proprioceptive, tactile, and visual feedback have been shown to promote neuroplasticity through enhanced functional connectivity within high-order transmodal networks, which are known to be influenced by circadian modulation of neural activity (43).

Exercise-induced neuroplasticity exemplifies the role of rhythmic motor activity in stroke recovery. Physical exercise enhances neurotrophin levels, synaptic remodeling, and interhemispheric connectivity, mechanisms that are tightly regulated by biological CLOCKS governing gene expression and neurotransmitter release (44). High-intensity interval training (HIIT), which imposes structured bouts of activity and rest, may further potentiate neuroplasticity by aligning with ultradian rhythms of neuronal excitability (45).

At the spinal level, transpinal direct current stimulation (tsDCS) has been reported to induce neuroplastic changes both locally and cortically, facilitating motor and cognitive recovery post-stroke. The effectiveness of tsDCS may be enhanced by timing stimulation to coincide with periods of heightened plasticity dictated by circadian rhythm (46). Similarly, interventions targeting intracortical and spinal reciprocal inhibition mechanisms, which are modulated by neurotransmitters such as GABA in a circadian manner, can influence functional motor recovery (47).

In summary, neuroplasticity is the cornerstone of stroke recovery, encompassing molecular, cellular, and network-level changes that restore impaired functions. Circadian rhythm acts as endogenous facilitators of neuroplasticity by regulating neuronal excitability, synaptic efficacy, and gene expression critical for neural remodeling. Therapeutic strategies that incorporate or mimic these rhythms-through environmental enrichment, timed neurostimulation, structured exercise, or multimodal feedback systems-hold promise for enhancing neuroplasticity and improving motor recovery outcomes in stroke patients. Future research should focus on elucidating the precise mechanisms by which circadian rhythm modulate neuroplasticity and on developing chronotherapy-based rehabilitation protocols tailored to individual patients’ circadian profiles.

### 2.3 The relationship between circadian rhythms and the recovery of athletic performance

#### 2.3.1 Mechanisms by which circadian rhythms regulate neural plasticity

Circadian rhythm, driven by molecular CLOCK genes, exert profound effects on neural plasticity, which is fundamental to motor recovery after stroke. The circadian system orchestrates daily oscillations in gene expression and protein networks that regulate neuronal excitability, synaptic remodeling, and neurogenesis—key processes underlying neural plasticity. For instance, core CLOCK genes such as CLOCK, Per1, Per2, Cry1, and Cry2 participate in the transcription-translation feedback loop (TTFL) that modulates neuronal signaling pathways and synaptic protein interactions. Experimental evidence from exercise models demonstrates that timing of physical activity influences the expression of these CLOCK genes and related proteomes in muscle and brain tissues, suggesting a time-of-day dependent modulation of neural circuits (48, 49). Specifically, rehabilitation after stroke selectively enhances synapse formation in neuronal populations projecting to affected motor areas, with parvalbumin interneurons playing a critical role in regulating neuronal connectivity and recovery of motor function (50). Parvalbumin interneurons contribute to gamma oscillations, a rhythm linked to synaptic synchronization and plasticity, which are directly modulated by circadian mechanisms. These oscillations are essential for long-term potentiation (LTP) and depression (LTD), the cellular basis of learning and memory that underpin motor skill acquisition and recovery. Moreover, circadian regulation of BDNF, a key mediator of synaptic plasticity, shows diurnal variation, with peak expression aligned with optimal windows for motor training (51, 52). At the molecular level, circadian rhythms regulate the expression of ion channels and neurotransmitter receptors, affecting synaptic strength and plasticity. CLOCK genes also influence the activation of microglia and astrocytes, which contribute to neuroinflammatory responses and subsequent plasticity after injury. MicroRNAs such as dmiR-283, whose expression is modulated by endurance exercise in a circadian manner, further link circadian rhythms to neural plasticity and motor control (53). The timing of exercise influences metabolic and proteomic profiles in the brain, with morning and evening exercise eliciting distinct molecular adaptations that may affect plasticity and recovery (48, 54). Taken together, these findings indicate that circadian rhythms regulate neural plasticity through complex mechanisms involving CLOCK gene-controlled neuronal activity, synaptic remodeling, interneuron function, and neurotrophic factor expression. Understanding these mechanisms can inform chronotherapeutic strategies to optimize rehabilitation by aligning interventions with patients’ intrinsic circadian rhythms, thereby enhancing neural plasticity and motor recovery.

#### 2.3.2 Analysis of relevant animal and clinical studies on circadian modulation of neural plasticity

**Figure 1:**
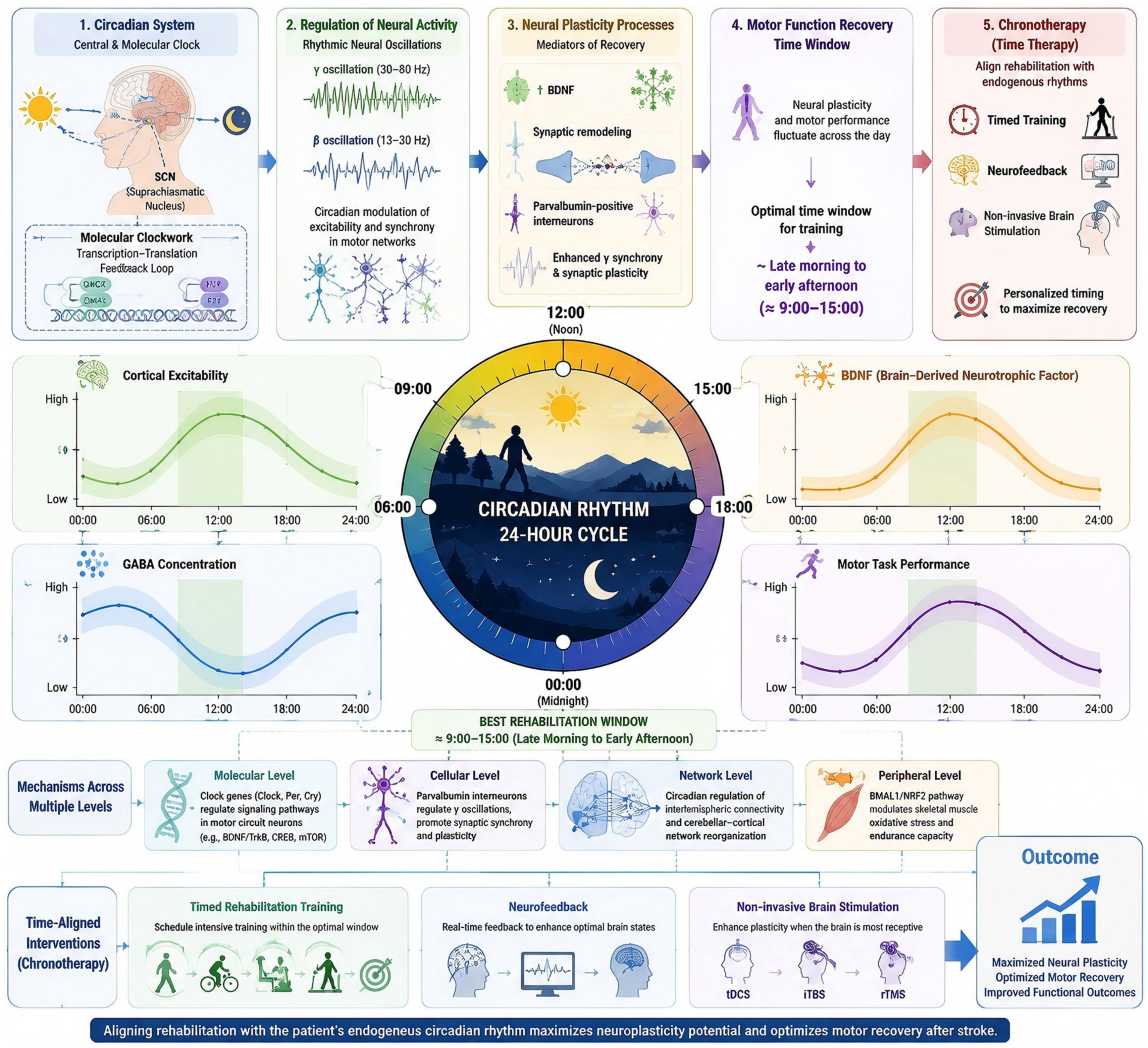
Circadian orchestration of motor recovery: from molecular clocks to chronotherapy. A multi-level mechanistic framework for circadian rhythm-based chronotherapy in post-stroke motor recovery. The schematic integrates regulatory mechanisms across multiple levels. (1) Circadian system: The central clock in the suprachiasmatic nucleus (SCN) generates ~24-h rhythms via a transcriptional-translational feedback loop involving core clock genes (e.g., CLOCK, BMAL1, PER, CRY). (2) Regulation of neural activity: these molecular oscillations drive rhythmic neural activity, including *γ* (30–80 Hz) and *β* (13–30 Hz) oscillations, which modulate cortical excitability and network synchrony in motor circuits. (3) Neural plasticity processes: circadian-regulated BDNF expression and parvalbumin-positive (PV+) interneuron activity promote synaptic remodeling and enhanced plasticity. (4) Motor function recovery time window: fluctuations in cortical excitability, GABAergic tone, and BDNF levels create a daily window of heightened plasticity, with the optimal period for motor training and rehabilitation occurring approximately from late morning to early afternoon (~9:00–15:00), when task performance peaks. (5) Chronotherapy: aligning rehabilitation interventions—including timed training, neurofeedback, and non-invasive brain stimulation—with this endogenous peak window maximizes neural plasticity, optimizes motor recovery, and improves functional outcomes after stroke.

The neuroplastic mechanisms orchestrated by the circadian system, as detailed above, suggest precise biological windows during which rehabilitation might be maximally effective. Before examining the supporting clinical and animal evidence, Figure 1 provides a schematic overview of these interconnected pathways, from the synchronization of central and peripheral clocks to the molecular and network-level determinants of recovery.

The integration of animal models and clinical studies reveals how circadian rhythms specifically influence neural plasticity mechanisms that drive motor recovery after stroke. In mice, rehabilitation enhanced synapse formation between presynaptic parvalbumin interneurons and postsynaptic neurons in motor areas, a process correlated with increased gamma oscillations and improved motor performance. Pharmacological enhancement of parvalbumin interneuron function mimicked these rehabilitation effects, underscoring the importance of circadian-regulated neuronal rhythmicity in driving synaptic plasticity. Studies have shown that the circadian clock controls the timing of synaptic protein synthesis and turnover, with key plasticity-related proteins such as Arc and c-Fos exhibiting rhythmic expression. This temporal regulation ensures that synaptic remodeling occurs during optimal recovery windows, directly impacting the effectiveness of rehabilitation training (50).

Complementing these mechanistic insights, clinical studies using neurofeedback approaches have demonstrated that modulating brain rhythms (e.g., beta rhythms) can enhance motor learning and plasticity in athletes, suggesting that entrainment of circadian-regulated neural oscillations may facilitate motor recovery (55). Advances in neuroimaging and brain-computer interface (BCI) technologies have further clarified the role of sensorimotor rhythms (SMRs) in neural plasticity. Optically pumped magnetometers (OPMs) capture SMRs during motor imagery, with real-time BCI systems enhancing plasticity by engaging sensorimotor networks through repeated rhythmic activation (56). This highlights the translational potential of rhythm-based neurotechnologies in promoting circadian-aligned neural plasticity for stroke rehabilitation.

Clinical investigations emphasize the connection between circadian rhythms, cognitive function, and plasticity-dependent motor learning. Rapid instructed task learning (RITL) is hindered after stroke due to impaired processing speed and inhibitory control-functions modulated by circadian rhythms. Disruptions in circadian rhythms can further impair cognitive speed and inhibition, limiting the brain’s capacity for activity-dependent plasticity. Studies indicate that motor recovery engages extensive brain networks integrating sensorimotor and attentional domains, with white matter disconnections influencing rehabilitation outcomes within rhythmic frameworks (57). Functional connectivity within motor networks, measurable by resting-state fMRI, predicts long-term motor recovery, with better interhemispheric functional connectivity-reflecting circadian-synchronized neural activity-predicting superior outcomes (58). Longitudinal studies reveal that functional connectome reorganization, particularly in cerebellar and subcortical networks, correlates with motor recovery, reflecting dynamic, rhythmic network adaptations post-stroke (59).

Rehabilitation interventions such as robot-assisted gait training have been shown to enhance activation in motor cortical areas and modulate electrophysiological markers like power ratio index and delta/alpha power ratio, which reflect underlying rhythmic activity. These neural changes parallel clinical improvements, reinforcing the importance of circadian-aligned cortical activity in driving plasticity (60). Non-invasive brain stimulation targeting the cerebellum, a key node in motor timing and rhythm generation, has demonstrated positive effects on gait and balance, further supporting the role of circadian rhythm in neural plasticity for rehabilitation (61).

Animal studies of perinatal stroke models also provide evidence that motor map size and movement latency, which are influenced by neuronal rhythmicity, serve as biomarkers for motor impairment and training responsiveness. Mice with smaller motor maps and worse impairments showed greater map expansion with skilled training, indicating that plasticity of rhythmic motor representations underlies recovery potential (62). Moreover, central fatigue, a phenomenon linked to disrupted neural rhythms, negatively impacts post-stroke motor recovery, with controlled peripheral fatigue mitigating central fatigue and enhancing motor outcomes in animal models (63).

Yuan et al. found that post-stroke mice trained during their active phase (ZT13) showed better motor recovery and increased neuronal survival, neurogenesis, and BDNF expression compared to those trained at rest (ZT6), supporting a circadian-aligned rehabilitation strategy (64).

Animal and clinical studies collectively demonstrate that circadian rhythms significantly influence post-stroke motor recovery by directly regulating neural plasticity mechanisms, including synaptic remodeling, neurotrophin expression, and functional network reorganization. Harnessing these rhythms through chronobiologically optimized rehabilitation, neurofeedback, and neuromodulation may enhance plasticity and recovery. Future research should focus on the temporal dynamics of these processes to develop personalized rehabilitation strategies aligned with individual circadian profiles.

A structured overview of the key studies discussed in this section, including their core design features, circadian measures, motor outcome metrics, and statistical findings, is presented in Table 1.

**Table 1:** Summary of key studies on circadian modulation of motor- and exercise-related outcomes.

Study	Sample size	Circadian measure	Motor outcome	Effect size (95% CI)	*p*-value
Human studies					
Sung et al. (75)	*N* = 70 subacute stroke	IS (7-day actigraphy)	BI at discharge	*β* = 0.23 (CI not reported); r = 0.46	*p* < 0.01
Dromerick et al. (76)	*N* ≈ 72 (adaptive randomization)	subacute (2–3 mo) vs. passive control	ARAT at 1 year (*Δ*, primary outcome)	Δ = +6.87 ± 2.63 (≈95% CI + 1.3 to +12.4)	*p* = 0.009
Dromerick et al. (76)	*N* ≈ 72	acute (≤30 d) vs. control	ARAT at 1 year (*Δ*)	Δ = +5.25 ± 2.59	*p* = 0.043
Dromerick et al. (76)	*N* ≈ 72	chronic (≥6 mo) vs. control	ARAT at 1 year (Δ)	Δ = +2.41 ± 2.25	*p* = 0.29
Červená et al. (77)	*N* = 30 healthy subjects	Chronotype (MEQ)	Cardio-pulmonary and HR recovery	N/R	*p* < 0.05
Pereira et al. (78)	*N* = 43 chronic stroke	Circadian chronotype by questionnaire	UE motor improvement after 10-day CIMT (MAL, AOU, QOM)	N/R	*p* < 0.05
Qian et al.(79)	*N* = 12 healthy adults	Circadian phase (core body temp)	Post-exercise systolic BP recovery	N/R	*p* = 0.029
Thomas et al.(80)	*N* = 52 young sedentary adults	DLMO (melatonin onset)	Circadian phase shift from timed exercise	0.62 h vs. − 0.02 h	*p* = 0.01
West et al. (81)	*N* = 43 (RCT, 2 arms)	IU vs. CU	Secondary to rehabilitation hospitalization (no direct motor outcome)	Median melatonin diff = 2.9 (IQR: −1.0 to 9.9)	*p* = 0.030 (levels); *p* = 0.007 (rhythmicity evolved)
Atanassova et al. (82)	*N* = 33 ischemic stroke + 33 matched controls	Nocturnal melatonin at 03:00 h (pg/ml)	Stroke presence probability (not direct motor outcome)	Adjusted OR = 1.020; 95% CI = 1.002–1.037 (per 1 pg./mL decrease)	*p* = 0.010
Atanassova et al. (82)	*N* = 66	Melatonin cut-off < 51.5 pg./mL	Stroke presence OR	OR = 3.12 (84% sensitivity, 74% specificity)	*p* = 0.0463
Nicolas et al.(83)	*N* = 11 healthy males	Time-of-day (06:00 vs. 18:00)	MVC & EMG recovery 2/3/7 days	N/R	N/R
Animal studies					
Yuan et al. (64)	Mice (3 groups)	ZT13 vs. ZT6 training	Motor recovery (cylinder test, grid walk)	N/R	*p* < 0.05 (ZT13 > ZT6)
Yuan et al. (64)	Mice	ZT13 vs. ZT20 training	Motor recovery	N/R	Trend only (ZT13 > ZT20)
Yuan et al. (64)	Mice	ZT13 vs. ZT6	Histological recovery markers	N/R	*p* < 0.05 (ZT13 > ZT6)
Zhu et al. (84)	Mice	Muscle stem cell clock	Muscle repair / inflammation	N/R	*p* < 0.05
Wolff et al. (85)	Mice	Exercise timing	Muscle clock phase shift	N/R	N/R
Reviews					
Hesketh et al.(86)	Review	Circadian timing and diurnal variation	time-of-day differences in exercise capacity	N/A	N/A
Martin et al. (87)	Review	Circadian muscle clock	Exercise performance patterns	N/A	N/A
Wolff et al. (88)	Review	Exercise timing and clock shifts	Exercise response patterns	N/A	N/A
Vitale et al. (89)	Review	Chronotype (M-type, E-type, N-type)	Athletic performance patterns	N/R	*p* < 0.05

N/R, not reported in the original study; N/A, not applicable; CI, confidence interval; ARAT, action research arm test; CIMT, constraint-induced movement therapy.

#### 2.3.3 Post-stroke motor recovery and circadian rhythms: neurofeedback as a modulatory strategy

Neurofeedback technology, a non-invasive method using real-time neural feedback (EEG, fMRI, NIRS), aids stroke rehabilitation by modulating brain activity to enhance motor recovery via neuroplasticity. It combines with motor imagery and BCI systems to strengthen motor network connectivity. Clinical studies show it can prevent cognitive decline and support motor function, though further research is needed to optimize protocols. Notably, it helps restore circadian rhythm-related oscillations disrupted after stroke.

Neurofeedback modulation of circadian rhythms enhances motor recovery post-stroke by realigning disrupted oscillations (e.g., gamma, beta, SMRs) linked to neuroplasticity. Key studies show targeting parvalbumin-regulated gamma and beta rhythms induces synaptic and physiological changes (50, 55, 56). Real-time SMR decoding using OPMs achieves >80% accuracy, aligning neurofeedback with circadian peaks (56). Cerebellar-cortical connectivity (e.g., lobule VIII, SMA) highlights rhythm-based therapy benefits (65). Rhythm-based therapies aligned with circadian rhythms, such as BCI training with targeted motor tasks and neurofeedback (66), mirror therapy (67), and soft robotic gloves (68), improve motor recovery after stroke by modulating theta-gamma coupling and cortical rhythms. These interventions enhance functional connectivity and synaptic plasticity, especially at optimal circadian times, underscoring rhythm-targeted neurofeedback as a personalized, circadian-aware strategy for post-stroke rehabilitation.

### 2.4 Future research directions and clinical applications

#### 2.4.1 Research challenges and opportunities

Research on the influence of circadian rhythm on motor recovery in stroke patients faces several critical challenges that limit the generalizability and applicability of findings. One major limitation is the relatively small sample sizes in many studies, which reduces statistical power and the ability to detect subtle but clinically meaningful effects. For instance, neuroimaging and neurophysiological studies often involve limited numbers of participants due to the complexity and cost of advanced imaging modalities like fMRI, EEG, or combined fNIRS-EEG systems, which restricts the robustness of conclusions about brain network reorganization and functional connectivity changes during recovery (69, 70). Small cohorts also hinder the exploration of heterogeneity among stroke patients, including variations in lesion location, severity, and individual circadian rhythm, which are crucial for personalized rehabilitation strategies.

Study design issues further complicate research in this field. Many investigations are observational or cross-sectional, limiting causal inferences about how circadian rhythm modulate motor recovery processes. Longitudinal designs, while more informative, require extensive resources and patient compliance over extended periods, which is challenging given the physical and cognitive impairments post-stroke. Moreover, the timing of assessments relative to stroke onset varies widely across studies, making it difficult to compare results or synthesize data. For example, some studies focus on acute or subacute phases, while others examine chronic stroke survivors, each with distinct neuroplasticity profiles (59, 71). This temporal variability affects the interpretation of biological rhythm influences and the identification of optimal intervention windows.

Another challenge lies in the heterogeneity of rehabilitation interventions and outcome measures. Motor recovery is assessed using diverse clinical scales (e.g., Fugl-Meyer Assessment, Action Research Arm Test), neurophysiological markers (e.g., cortico-cortical correlation coefficients), and neuroimaging parameters (e.g., functional connectivity, muscle synergy indices), which complicates cross-study comparisons and meta-analyses (72, 73). Additionally, many studies rely on subjective or semi-quantitative assessments, which may introduce bias or variability. The lack of standardized protocols for measuring and integrating biological rhythm parameters, such as circadian phase or sleep–wake cycles, further limits the ability to elucidate their mechanistic roles in motor recovery.

Technological limitations also present obstacles. While advances such as OPMs enable real-time monitoring of SMRs with fewer sensors, their application in clinical stroke populations remains in early stages and requires validation (56). Similarly, the integration of multimodal neuroimaging techniques, like combined EEG-fNIRS, offers promising avenues for detailed characterization of neurovascular coupling and cortical reorganization but demands sophisticated data processing and interpretation frameworks (70). These complexities may restrict widespread adoption and reproducibility.

Significant opportunities exist in advancing the field through machine learning models that utilize clinical data for predicting motor recovery, enabling individualized rehabilitation plans aligned with circadian rhythms. Novel therapeutic approaches combining pharmacological neuromodulation with exercise have shown enhanced recovery, suggesting that timing interventions can optimize outcomes. The understanding of specific neuronal populations, like parvalbumin interneurons, opens new pharmacological targets modulated with biological cycles (74).

Current research shows methodological limitations, including small samples and variable outcomes, hindering conclusions on circadian rhythms’ impact on post-stroke recovery. However, advances in neuroimaging and combined therapies suggest promising solutions, necessitating future studies with larger cohorts and standardized assessments for effective rehabilitation.

As the systematic synthesis in Table 1 illustrates, a common and critical limitation across the current literature is the pervasive under-reporting of effect sizes and associated confidence intervals. Of the primary studies summarized, the majority did not provide these precision estimates—a gap denoted as ‘N/R’ (Not Reported) in the table. This reporting deficit represents a significant barrier to conducting robust meta-analyses and to deriving clinically meaningful effect magnitude estimations, underscoring the need for standardized reporting guidelines in future chronobiological rehabilitation research.

#### 2.4.2 Clinical application prospects

Modulating circadian rhythm shows potential for improving stroke rehabilitation, enhancing motor recovery through specific neuronal circuits and rhythmic activity. Research in mice indicates rehabilitation fosters synapse formation between parvalbumin interneurons and motor area neurons, correlating with better motor performance. Parvalbumin interneurons, crucial for recovery, require activation during training, and pharmacological enhancement of their function mimics rehabilitation effects, suggesting new therapeutic targets to aid traditional strategies in stroke recovery (50).

Neurobiofeedback techniques, such as beta rhythm modulation, show therapeutic potential in various fields like sports medicine by inducing cardiovascular changes in athletes, which may enhance motor control and endurance. While studies focus on healthy athletes, rhythm-based feedback could aid stroke rehabilitation by improving neural plasticity and motor recovery, highlighting the need for personalized clinical approaches (55).

Future research should develop rhythm-targeted rehabilitation combining physical therapy with neurofeedback or pharmacological agents to enhance interneuron function. Clinical trials must validate the efficacy and safety of these interventions across stroke populations, considering factors like stroke severity and individual biological rhythms. Understanding how rhythm modulation affects synaptic plasticity and motor recovery will support rational drug design. Additionally, non-invasive techniques synchronized with endogenous rhythms or patient-specific neurofeedback training present promising opportunities. The integration of neurophysiological insights with rhythm-based interventions can significantly improve outcomes for stroke survivors.

## 3 Conclusion

In conclusion, the exploration of circadian rhythm in the context of motor function recovery following stroke represents a promising and evolving frontier in neurorehabilitation. This review underscores the critical role that circadian and other endogenous rhythms play in modulating neural activity and facilitating neuroplasticity, which are foundational processes for restoring motor abilities in stroke survivors. The integration of chronobiological principles into stroke rehabilitation not only broadens our understanding of the temporal dynamics underlying neural recovery but also opens novel avenues for optimizing therapeutic interventions.

Recent evidence, as synthesized in the mechanistic framework of Figure 1, demonstrates that post-stroke recovery in animals improves significantly when rehabilitation aligns with their active circadian phase, enhancing synaptic plasticity and neuronal survival (64). These converging findings, from molecular clock gene oscillations to network-level interhemispheric connectivity, demonstrate that timing is a critical, accessible, and no-cost variable.

Balancing the diverse research perspectives, it is evident that while traditional rehabilitation approaches have primarily focused on task-specific training and physical therapy, incorporating the timing of interventions aligned with patients’ circadian rhythm may enhance efficacy. As the quantitative evidence summarized in Table 1 demonstrates, interventions timed to coincide with peak periods of circadian-regulated plasticity consistently yield superior motor outcomes across both human and animal studies. However, the heterogeneity of stroke pathology and individual differences in circadian patterns, and the incomplete reporting of effect sizes highlighted in Table 1 present challenges that necessitate both personalized rehabilitation protocols and more standardized research practices.

Moreover, the advent of neurofeedback technologies offers an exciting opportunity to harness circadian rhythm in real-time, enabling dynamic modulation of neural circuits involved in motor control. Neurofeedback interventions, tailored to the patient’s intrinsic circadian rhythm, could potentiate neuroplastic changes and accelerate functional gains. However, the existing body of evidence is still quite restricted, and comprehensive clinical studies are necessary to confirm these methods and develop standardized protocols.

Future research should prioritize elucidating the molecular and cellular mechanisms through which circadian rhythm influence motor recovery after stroke. Understanding how circadian genes and clock-controlled pathways interact with neuroplastic processes will be crucial for developing targeted therapies. Additionally, interdisciplinary collaboration integrating chronobiology, neuroscience, and rehabilitation medicine will be essential to translate these insights into clinical practice effectively.

In summary, the incorporation of biological rhythm considerations into stroke rehabilitation represents a paradigm shift that holds significant potential to improve motor function recovery. By aligning existing rehabilitation strategies with the chronobiological mechanisms outlined in Figure 1 and the clinical evidence catalogued in Table 1, clinicians can move toward more personalized and effective treatment regimens. Continued investigation in this domain promises to refine our approaches and ultimately enhance the quality of life for stroke survivors through optimized, chronobiologically-informed neurorehabilitation.
